# Major Depressive Disorder and Driving Behavior Among Older Adults

**DOI:** 10.1001/jamanetworkopen.2024.52038

**Published:** 2024-12-30

**Authors:** Ganesh M. Babulal, Ling Chen, Jean-Francois Trani, David C. Brown, David B. Carr, Beau M. Ances, Eric J. Lenze

**Affiliations:** 1 Department of Neurology, Washington University in St Louis, St Louis, Missouri; 2 Institute of Public Health, Washington University in St Louis, St Louis, Missouri; 3 Department of Psychology, Faculty of Humanities, University of Johannesburg, Johannesburg, South Africa; 4 Division of Biostatistics, Washington University in St Louis, St Louis,Missouri; 5 Brown School of Social Work, Washington University in St Louis, St Louis, Missouri; 6 National Conservatory of Arts and Crafts, Paris, France; 7 Department of Medicine, Washington University in St Louis, St Louis, Missouri; 8 Department of Psychiatry, Washington University in St Louis, St Louis, Missouri

## Abstract

**Importance:**

Depression and antidepressant use are independently associated with crash risk among older drivers. However, it is unclear what factors impact daily driving that increase safety risk for drivers with depression.

**Objective:**

To examine differences in naturalistic driving behavior and safety between older adults with and without major depressive disorder (MDD).

**Design, Setting, and Participants:**

A prospective longitudinal cohort study was conducted among older adults (≥65 years) from the Driving Real-World In-Vehicle Evaluation System Project collected from July 1, 2021, to December 30, 2023. The sample included 85 participants with MDD and 310 participants without. Neurological, clinical, mood, and neuropsychological tests were collected annually. Daily driving behavior was recorded using a commercial data logger. Statistical analysis was performed from January 31 to June 24, 2024.

**Exposure:**

MDD and antidepressant usage.

**Main outcomes and measures:**

Linear mixed models with propensity score weighting compared slopes of driving behaviors over time (trips taken at night, speeding, hard braking, entropy, and radius of gyration) between groups.

**Results:**

In a sample of 395 participants, 85 were classified as individuals with MDD (mean [SD] age, 69.6 [6.1] years; 60 [70.6%] female; 8 [9.4%] non-Hispanic Black and 77 [90.6%] non-Hispanic White) and 310 as individuals in the control group without depression (mean [SD] age, 70.1 [5.1] years; 153 [49.4%] female; 40 [12.9%] non-Hispanic Black and 270 [87.1%] non-Hispanic White). Adults with MDD had greater depressive symptoms (mean [SD], 8.35 [5.35] vs 2.33 [2.72]; difference, 6.02; 95% CI for difference, 5.17 to 6.85; *P* < .001), comorbidities (mean [SD], 4.08 [2.07] vs 2.79 [1.67]; difference, 1.29; 95% CI for difference, 0.87 to 1.70; *P* < .001), used more antidepressants (mean [SD], 0.94 [0.81] vs 0.27 [0.54]; χ^2^_1_ = 65.8; *P* < .001), and had a higher number of medications (mean [SD], 3.80 [3.27] vs 1.98 [2.21]; χ^2^_1_ = 21.0; *P* < .001) compared with controls at baseline. Longitudinal analysis demonstrated an association between adults with MDD and hard braking (mean [SE], 3.17 × 10^−4^ [7.30 × 10^−5^] vs 6.70 × 10^−5^ [4.00 × 10^−5^]; difference, 2.50 × 10^−4^; 95% CI for difference, 1.74 × 10^−4^ to 4.61 × 10^−4^; *P* < .001) and hard cornering events per trip (mean [SE], 0.80 [0.64] vs 0.57 [0.25]; difference, 0.23; 95% CI for difference, 0.08 to 1.06; *P* = .04), greater distances driven from home (mean [SE], 31.19 [7.35] vs 7.76 [3.80] km; difference, 23.43; 95% CI for difference, 0.28 to 15.2; *P* < .001), more unique destinations visited (mean [SE], 0.34 [0.10] vs −0.27 [0.03]; difference, 0.61; 95% CI for difference, 0.14 to 0.54; *P* < .001), and higher random entropy (mean [SE], 0.01 [0.01] vs −0.02 [0.00]; difference, 0.03; 95% CI for difference, −0.03 to −0.01; *P* < .001) over time.

**Conclusions and relevance:**

In this longitudinal cohort study of older drivers, adults with MDD demonstrated distinct and riskier driving behaviors than those in the control group without depression, with higher rates of hard braking, cornering, and unpredictability in driving patterns over time. Routine depression screening and tailored interventions are essential for enhancing driving safety and maintaining independence among older adults with MDD. Comprehensive care approaches addressing both mental and physical health are crucial for this vulnerable population.

## Key Points

**Question:**

After adjustment for medications that impair driving, is major depressive disorder (MDD) associated with driving decline among older adults?

**Findings:**

In this cohort study with 395 participants, older adults with MDD exhibited riskier driving behaviors, such as more frequent hard braking, hard cornering, and unpredictable driving patterns, compared with individuals without depression. There were no differences in cognitive functioning at baseline.

**Meaning:**

These findings suggest that MDD is associated with driving safety among older adults, highlighting the need for targeted interventions to help them maintain safe driving habits and independence.

## Teaser

This cohort study evaluates differences in naturalistic driving behavior and safety between older adults with and without major depressive disorder.

## Introduction

By 2050, the older adult population (aged ≥65 years) will reach 88 million in the US and 400 million worldwide.^1^ An individual aged 65 years can expect to live 19 or more years, and an individual aged 85 years can expect to live, on average, another 6 to 7 years.^2^ It is also estimated that 25% of all drivers will be older than 65 years by 2050.^3^ Driving mobility is critical since many older adults are not retiring at the traditional age of 65 but remain employed in full- or part-time jobs past 70 years old.^4^ Driving provides social participation and access to health care services, facilitating autonomy, independence, and the ability to age in place. Older drivers prefer to drive themselves or be transported in a personal vehicle even when options are available (eg, mass transit or e-hail services).^5^ A greater understanding of common factors that lead to changes in driving behavior with aging is needed.

A meta-analysis (72 878 participants across 48 studies) found a 28% global prevalence of depression among older adults.^6^ In the US, depression (13% prevalence in 2022) is common, with a higher rate among women compared with men.^7^ Major depressive disorder (MDD) affects approximately 8.4% of adults (aged ≥18 years) in the US.^8^ Late-life depression refers to MDD in older adults; it includes lifelong recurrent illness and late-onset depression stemming from biological risks (eg, biological decline and/or comorbidities or acute conditions like stroke) and psychosocial risks (eg, bereavement of family and/or friend deaths or change in income).^9^ Late-life depression often goes undiagnosed.^10^ A 2022 survey found that only 39.4% of individuals had a formal depression diagnosis; however, only 53% considered seeking mental health expertise, and 31% of respondents without a formal diagnosis had moderate depression.^11^

Compared with younger drivers (aged 25-64 years), older drivers are more likely to cause a crash.^12^ Since older adults are driving longer, the prevalence of crashes and resulting deaths to drivers or pedestrians will increase based on the volume of active drivers. An increased crash risk among aging drivers has been attributed to different factors, including medication adverse effects, cognitive decline associated with dementia, and depression.^13^ Late-life depression shares common impairments in cognitive performance, such as a decline in reaction time, divided attention, executive function, and short-term memory.^14^ Impaired cognitive processing as a consequence of depression is thought to be responsible for associations between depression, poorer driving performance, and higher crash risk.^15^ Using a combination of the Geriatric Depression Scale, General Health Questionnaire, interviews, and claims-based databases to identify depression, a meta-analysis established depression as nearly doubling the odds of crash, irrespective of age (OR, 1.90; 95% CI, 1.06-3.39).^16^ A prior study^17^ from our group found that among a cohort of cognitively normal older adults, those with depression were 3 times more likely (HR, 3.20; 95% CI, 1.48-6.95) to receive a marginal or failing rating on a standardized road test over a 2.5-year follow-up period.

Studies examining fitness to drive (controlled conditions) and crash risk (based on claims data) are primarily conducted in healthy samples without depression.^18^ This longitudinal cohort design examines whether older adults with MDD experience a more significant decline and changes using in vivo daily driving behavior compared with those without depression (characterized by diagnosis or symptoms). We hypothesize that older adults with MDD will experience greater changes in naturalistic driving behavior over time, exhibit risky behaviors like more speeding and hard braking, visit fewer destinations, and have a smaller number of trips compared with nondepressed, healthy participants.

## Methods

### Participant Sample

All participants were enrolled in a prospective longitudinal cohort study, the Driving Real-World In-Vehicle Evaluation System (DRIVES) Project at Washington University School of Medicine, that examined how aging changes are associated with complex neurobehavioral activities such as driving. Participants in the control group were required to be aged at least 65 years or older and cognitively normal at baseline as determined by a 0 on the Clinical Dementia Rating (CDR).^19^ Participants in the MDD group were required to be aged 65 years or older, have an MDD diagnosis by a clinician and/or have a Patient Health Questionnaire (PHQ-9) score of 10 or more and score a 0 or 0.5 on the CDR. Participants were not eligible for the study if they had a previous diagnosis of memory impairment that was unrelated to depression. Recruitment strategies included flyers, social media, clinic referrals, and Epic electronic health records. Data on race and ethnicity were collected at the first visit, based on self-reported ethnicity (Hispanic or non-Hispanic) and racial categories (African American/Black, and White). Race and ethnicity were assessed because they are proxies for social determinants of health, and individuals racialized as Black and Hispanic have a higher risk of depression compared with non-Hispanic White individuals. Data on sex was self-reported based on 2 options (male and female). All recruitment, patient-provided written informed consent, and study protocols were approved by the Washington University School of Medicine institutional review board. This prospective cohort study followed the Strengthening the Reporting of Observational Studies in Epidemiology (STROBE) reporting guideline.

#### Clinical and Neuropsychological Battery

The CDR was completed by each participant and their respective collateral source. The global CDR (0-3) and CDR-sum of boxes (CDR-SB) (0-18) were derived from scores across memory, orientation, judgment and problem-solving, community affairs, home and hobbies, and personal care domains. No participants had moderate or severe dementia. The CDR consensus evaluation incorporated sociodemographic (eg, age, education, and socioeconomic status) and clinical factors based on the original procedures.^19^ Participants completed several neuropsychological assessments, including the Trail Making Test A and B, Category Fluency, Verbal Fluency, and Free and Cued Selective Reminding Task: Free Recall Score. A baseline cognitive composite^20^ score was computed by standardizing the scores of the latter 4 subtasks using their means and SDs and then calculating the mean of each participant’s standardized scores. The PHQ-9,^21^ based on the *Diagnostic and Statistical Manual of Mental Disorders* (Fourth Edition) criteria for major depressive disorder, screens for depressive symptoms over the past 2 weeks with 9 items with higher scores (range 0-27) indicating higher levels of depressive symptoms. The Subjective Happiness Scale (SHS),^22^ a 4-item scale, assessed overall happiness, with higher scores (range 1-7) indicating greater happiness. From a review of medical history, a count of 15 medical conditions (arthritis, diabetes, hypertension, hyperlipidemia, B_12_ deficiency, thyroid disorder, angiopathy, congestive heart failure, atrial fibrillation, urinary inconstancy, fecal incontinence, sleep apnea, rapid eye movement behavior disorder, insomnia, and other sleep disorders) measured comorbidity.

### Medications

Medication data were collected at baseline visit per the National Alzheimer Coordinating Center Uniform Data Set guidelines via self-reported medication taken within the past 2 weeks. The American Hospital Formulary Service^23^ classification system grouped all prescribed and over-the-counter medications into classes that reflect similar pharmacologic and therapeutic characteristics. All drug classes were reviewed to identify potentially driver-impairing medication categories as defined by the US Food and Drug Administration^24^ and previous studies.^25^ A comprehensive list of all medications, their respective classification system, and 8 categories are available (eTable 1 in Supplement 1).

### Naturalistic Driving Data Collection

A commercial vehicle data logger (Azuga, G2) was plugged into each participant’s personal vehicle’s onboard diagnostic port. Daily driving behavior was continuously collected from ignition start to ignition off. Tabular data included date, time, speed, latitude, and longitude, along with event-based threshold alerts for speeding or hard braking. DRIVES method examined metrics, including the total number of trips, the average distance traveled, trips taken at night, speeding, hard braking, hard cornering (lateral acceleration exceeding 0.4 g force), entropy (degree of predictability between locations visited), and radius of gyration (speed of turning and steering relating to stability), among others (see eTable 3 in Supplement 1).^26,27^ Data were aggregated daily for each participant, processed, and cleaned to summarize any specific behavior over any epoch.

### Statistical Analysis

Driving data were truncated to include data from July 1, 2021, to December 30, 2023, for comparable follow-up time between groups. Analyses assumed a linear association between aggregated monthly naturalistic driving variables and time. A random coefficients model (linear mixed model) estimated the average rate of change in driving outcomes across groups allowing y-intercepts and slopes (monthly rate of change) to vary randomly between participants and fitted a separate regression line for each participant. The y-intercept estimated the mean of a driving variable at the beginning of data collection (baseline). To adjust for the imbalance between the sample size of the 2 groups and account for unmeasured confounders, we calculated a propensity score weight^28^ using logistic regression. The analysis provided a weight for each participant across groups, as the probability of the treatment (depression) assignment was conditional on self-reported age, sex, race, and education. The propensity score was used to compute the inverse probability of treatment weighting (IPTW)^29^ in the random coefficients model analysis. The analyses using random coefficient models adjusted for depression status, age, sex, race, education, and the number of medical conditions. The interaction between case and control groups and time was considered to test for differences in slope over time, and the model examined whether there was a difference in the y-intercept. Secondary analyses included an antidepressant composite, while another included total number of medications. Estimated means of the driving variables of each participant were obtained from the random coefficients model, and locally estimated scatter plot smoothing was applied to visualize the y-intercept and slope change over time between groups. All statistical tests were 2-tailed at a significance level of .05, and statistical analysis was performed with SAS 9.4 (SAS Institute). Data were analyzed from January 31 to June 24, 2024.

## Results

In a sample of 395 participants, 85 were classified as individuals with MDD (mean [SD] age, 69.6 [6.1] years; 60 [70.6%] female; 8 [9.4%] non-Hispanic Black and 77 [90.6%] non-Hispanic White) and 310 as individuals in the control group without depression (mean [SD] age, 70.1 [5.1] years; 153 [49.4%] female; 40 [12.9%] non-Hispanic Black and 270 [87.1%] non-Hispanic White). There were no significant differences in age, years of education, or race. However, there was a statistically significant difference in sex, with a larger proportion of women among those with depression (Table 1). Compared with the control group, participants with MDD, on average, had more comorbid conditions (mean [SD], 4.08 [2.07] vs 2.79 [1.67]; difference, 1.29; 95% CI for difference, 0.87 to 1.70; *P* < .001). Additionally, participants with MDD had slightly higher scores on the CDR-SB (mean [SD], 0.91 [0.91] vs 0.45 [1.07]; difference, 0.46; 95% CI for difference, 0.22 to 0.71; *P* < .001) at baseline compared with those in the control group, resulting in a larger proportion of participants with CDR greater than 0, indicating cognitive impairment. While there were no statistically significant differences in any of the neuropsychological tests or the cognitive composite between those with MDD and those without, those with MDD performed worse. Participants with MDD had more depressive symptoms (PHQ-9: mean [SD], 8.35 [5.35] vs 2.33 [2.72]; difference, 6.02; 95% CI for difference, 5.17 to 6.85; *P* < .001) and lower happiness (SHS: mean [SD], 4.34 [1.34] vs 5.65 [1.01]; difference, −1.31; 95% CI for difference, −1.59 to −1.05; *P* < .001) compared with the control group. Those in the control group had their data logger plugged into their vehicles for a mean (SD) of 1.91 (0.43) years from the CDR compared with 1.12 (0.36) years for those with MDD (difference, 0.79; 95% CI for difference, 0.63 to 0.83; *P* < .001). Participants with MDD self-reported taking more medications across 7 individual categories, both in the antidepressant composite (mean [SD], 0.94 [0.81] vs 0.27 [0.54]; χ^2^_1_ = 65.8; *P* < .001) and in the total number of medications (mean [SD], 3.80 [3.27] vs 1.98 [2.21]; χ^2^_1_ = 21.0; *P* < .001). There was no difference in tricyclic antidepressant use and no participants reported taking monoamine oxidase inhibitors (Table 1).

**Table 1.** Participant Characteristics at Baseline

Characteristic	Participants, mean (SD)		*P* value
	With MDD (n = 85)	Without MDD (n = 310)	
Age, y	69.59 (6.09)	70.38 (5.13)	.24
Education, y	16.55 (2.34)	16.35 (2.44)	.50
Sex, No. (%)			
Female	60 (70.6)	153 (49.4)	<.001
Male	25 (29.4)	157 (50.6)	
Race and ethnicity, No. (%)			
Non-Hispanic Black	8 (9.4)	40 (12.9)	.38
Non-Hispanic White	77 (90.6)	270 (87.1)	
CDR at DRIVES installation, No. (%)			
0	40 (47.1)	242 (78.1)	<.001
0.5	44 (51.8)	62 (20.0)	
1	1 (1.2)	6 (1.9)	
CDR sum of boxes	0.91 (0.91)	0.44 (0.24)	.001
TMT A	−0.02 (0.99)	0.08 (1.06)	.45
TMT B	0.022 (1.04)	−0.08 (0.86)	.37
VF Animals	−0.05 (1.04)	0.16 (0.83)	.06
SRT Free	−0.03 (1.05)	0.09 (0.79)	.27
Cognitive composite	−0.02 (0.49)	0.06 (0.42)	.14
Follow-up time, d	14.15 (42.27)	102.07 (116.22)	<.001
Time from chip install to CDR, d	2.73 (3.38)	3.01 (3.58)	<.001
PHQ-9	8.35 (5.35)	2.33 (2.72)	<.001
SHS	4.36 (1.34)	5.66 (1.01)	<.001
Comorbidities	4.08 (2.07)	2.79 (1.67)	<.001
Medications			
SSRI/SNRI	0.67 (0.68)	0.16 (0.39)	<.001
TCA	0.02 (0.15)	0.02 (0.13)	.67
Antipsychotics	0.09 (0.33)	0.01 (0.01)	<.001
Benzodiazepines	0.15 (0.42)	0.03 (0.16)	<.001
Anticonvulsants	0.18 (0.38)	0.09 (0.29)	.03
Opioids	0.09 (0.33)	0.01 (0.16)	.002
NSAID/Acetaminophen	0.43 (0.81)	0.57 (0.72)	.02
CNS-D	2.08 (1.96)	1.07 (1.19)	<.001
Antidepressant composite	0.94 (0.81)	0.27 (0.54)	<.001
Total composite	3.80 (3.27)	1.98 (2.21)	<.001

Abbreviations: CDR, clinical dementia rating; CNS-D, central nervous system drugs; DRIVES, Driving Real-World In-Vehicle Evaluation System; MDD, major depressive disorder; NSAID, nonsteroidal anti-inflammatory drug; PHQ-9, Patient Health Questionaire-9; TMT A, Trailmaking Test A; TMT B, Trailmaking Test B; SHS, Subjective Happiness Scale; SRT Free, Selective Reminding Task, Free Recall; SSRI/SNRI, selective serotonin reuptake inhibitor/serotonin norepinephrine reuptake inhibitor; TCA. tricyclic antidepressant; VF, verbal fluency;.

When examining behavior at baseline (Table 2), compared with those in the control group, older adults with MDD had a higher number of speeding events (mean [SE], 37.50 [33.29] vs 20.09 [32.02]; χ^2^_3.95_ = 185; *P* = .04), more hours driven per month (mean [SE], 54.90 [9.94] vs 50.80 [9.69] hours; χ^2^_3.95_ = 212; *P* = .04), and a slightly higher mean trip time (mean [SE], 21.49 [4.05] vs 19.27 [3.93] minutes; χ^2^_5.65_ = 131; *P* = .02). There were no statistically significant differences in adverse driving behaviors like braking, sudden acceleration, or cornering. Additionally, there were no significant group differences in spatial distances by trips or measures of dispersion.

**Table 2.** Driving Behavior Outcomes Across Drivers With and Without Major Depressive Disorder (MDD), Where Each Variable Represents the Mean Number per Trip or Summary per Month

Naturalistic driving	Y-intercept			Slope		
	Mean (SE)		*P* value	Mean (SE)		*P* value
	Without MDD	With MDD		Without MDD	With MDD	
Hard braking	5.80 × 10^−4^ (4.46 × 10^−3^)	7.80 × 10^−4^ (4.55 × 10^−3^)	.13	6.70 × 10^−5^ (4.00 × 10^−5^)	3.17 × 10^−4^ (7.30 × 10^−5^)	<.001
Sudden acceleration	0.02 (0.03)	0.01 (0.03)	.30	0.00 (1.22 × 10^−4^)	2.08 × 10^−4^ (2.15 × 10^−4^)	.34
Speeding	20.09 (32.02)	37.50 (33.29)	.04	−0.18 (0.14)	−0.17 (0.27)	.38
Hard cornering	31.73 (21.03)	30.78 (21.93)	.88	0.57 (0.25)	0.80 (0.64)	.04
Trip distance						
1 to 5 Miles	62.69 (21.11)	66.07 (21.63)	.41	0.00 (0.03)	−0.19 (0.05)	<.001
5 to 10 Miles	25.42 (5.93)	25.72 (6.07)	.78	0.01 (0.03)	0.02 (0.06)	.87
10 to 20 Miles	31.47 (5.82)	32.02 (5.97)	.63	0.00 (0.02)	−0.01 (0.04)	.94
Hours driven	50.80 (9.69)	54.90 (9.94)	.04	0.00 (0.05)	−0.12 (0.08)	.37
Trip time, min	19.27 (3.93)	21.49 (4.05)	.02	−0.01 (0.02)	−0.06 (0.03)	.19
Days driven per month	28.47 (3.70)	29.02 (3.78)	.42	−0.02 (0.02)	−0.08 (0.03)	.01
Radius of gyration	14.33 (44.57)	16.92 (46.01)	.83	0.63 (0.54)	3.11 (1.04)	.01
Maximum distance from home, km	125.09 (228.89)	81.35 (235.31)	.51	7.76 (3.80)	31.19 (7.35)	<.001
Maximum distance driven, km	101.00 (206.87)	44.02 (212.63)	.36	9.00 (3.81)	32.33 (7.38)	<.001
Random entropy	5.11 (0.62)	5.32 (0.64)	.10	−0.02 (0.00)	0.01 (0.01)	<.001
Destinations visited	41.23 (12.93)	44.03 (13.30)	.28	−0.27 (0.05)	0.34 (0.10)	<.001

**Figure 1.**
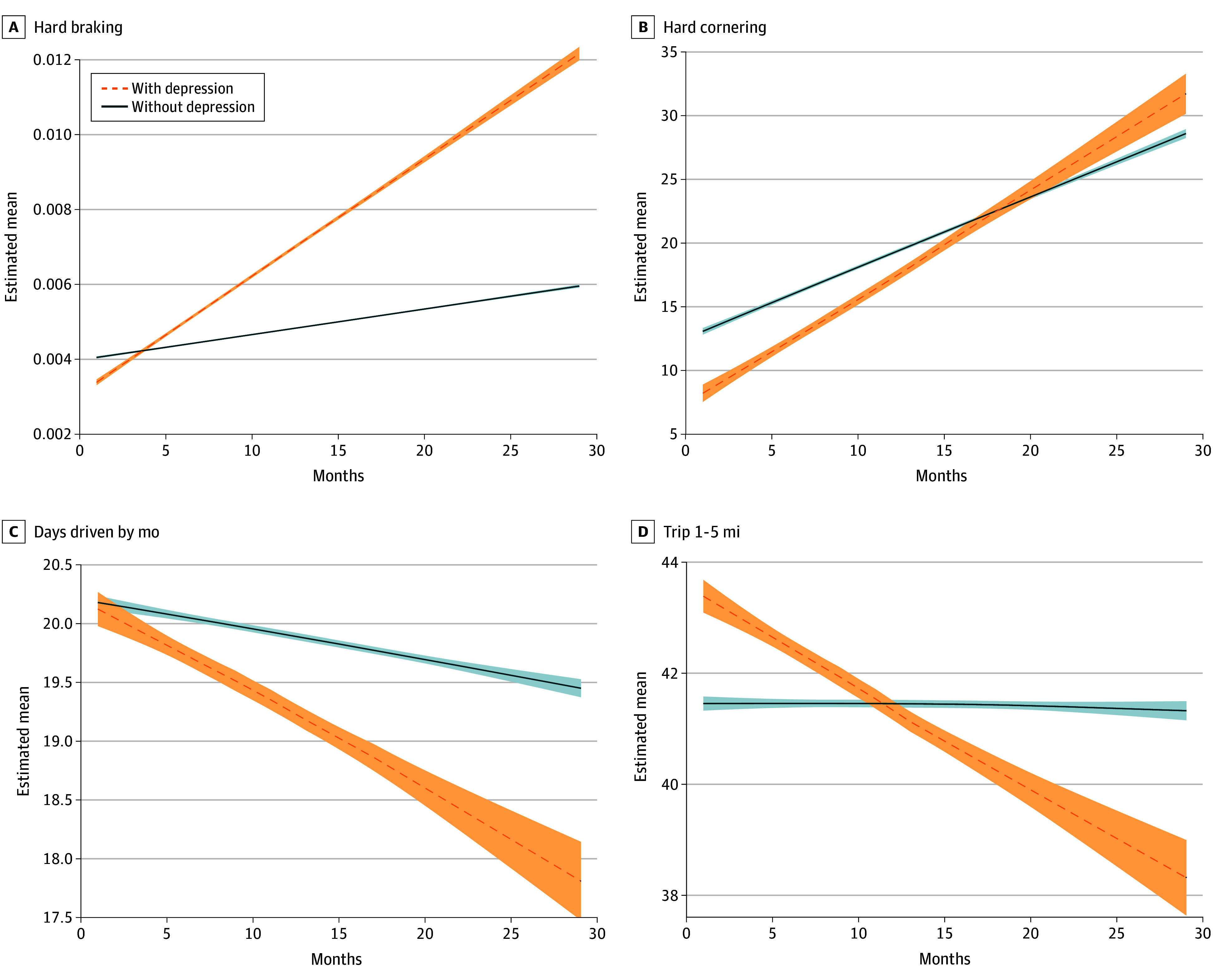
Driving Outcomes Across Adverse Events and Driving Frequency Shaded areas represent the 95% CI for the fitted curves corresponding to each group.

**Figure 2.**
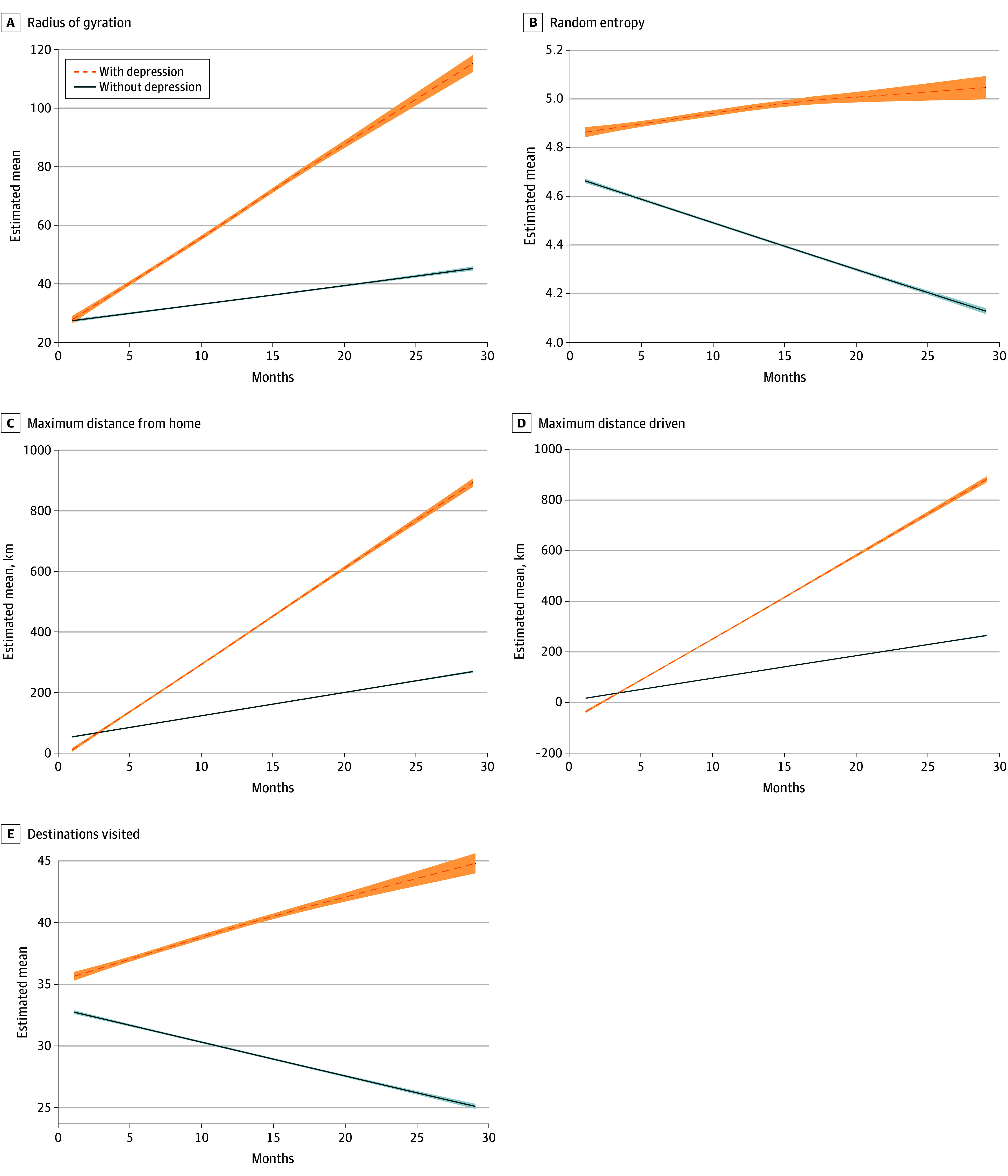
Driving Outcomes in Dispersion and Destination Visited Shaded areas represent the 95% CI for the fitted curves corresponding to each group.

Longitudinally, drivers with MDD were associated with a greater number of hard braking events (mean [SD], 3.17 × 10^−4^ [7.30 × 10^−5^] vs 6.70 × 10^−5^ [4.00 × 10^−5^]; difference, 2.50 × 10^−4^; 95% CI for difference, 1.74 × 10^−4^ to 4.61 × 10^−4^; *P* < .001) and hard cornering events based on mean events per trip (mean [SD], 0.80 [0.64] vs 0.57 [0.25]; difference, 0.23; 95% CI for difference, 0.08 to 1.06; *P* = .04) (Table 2). Conversely, drivers with MDD showed a decrease in trips driven between 1 and 5 miles and days driven per month (Figure 1). There were divergent slopes with random entropy (mean [SE], 0.01 [0.01] vs −0.02 [0.00]; difference, 0.03; 95% CI for difference, −0.03 to −0.01; *P* < .001) and radius of gyration, where participants with MDD were associated with more risky driving behaviors over time and compared with the control group (Figure 2). Additionally, there were differences in the slopes between groups on the overall maximum distance driven, distance traveled from their home (mean [SD], 31.19 [7.35] vs 7.76 [3.80] km; difference, 23.43; 95% CI for difference, 0.28 to 15.2; *P* < .001), and the number of destinations visited (mean [SD], 0.34 [0.10] vs −0.27 [0.50]; difference, 0.61; 95% CI for difference, 0.14 to 0.54; *P* < .001). When the models were adjusted for any antidepressant use, all the models remained statistically significant (Table 3). In fact, the y-intercept random entropy model became statistically significant (mean [SD], 5.43 [0.62] vs 5.15 [0.60]; difference, 0.28; 95% CI for difference, −0.40 to −0.01; *P* = .03), suggesting that drivers with MDD had a higher dispersion of entropy at baseline. Finally, when substituting antidepressant use for total medication use (across 8 categories), the results remained unchanged (eTable 2 in Supplement 1).

**Table 3.** Driving Behavior Outcomes Across Drivers With and Without Major Depressive Disorder (MDD), Where Each Variable Represents the Mean Number per Trip or Summary per Month With Addition of Antidepressant Use

Naturalistic driving	Y-intercept			Slope		
	Mean (SE)		*P* value	Mean (SE)		*P* value
	Without MDD	With MDD		Without MDD	With MDD	
Hard braking	2.00 × 10^−3^ (4.30 × 10^−3^)	1.10 × 10^−3^ (4.40 × 10^−3^)	.30	1.00 × 10^−4^ (4.00 × 10^−5^)	3.00^−4^ (1.00 × 10^−4^)	<.001
Sudden acceleration	0.01 (0.03)	0.01 (0.03)	.20	1.00 × 10^−4^ (1.00 × 10^−4^)	2.00 × 10^−4^ (2.00 × 10^−4^)	.20
Speeding	24.39 (32.22)	46.74 (33.77)	.02	−0.17 (0.14)	−0.17 (0.27)	.42
Hard cornering	32.97 (21.24)	30.78 (22.20)	.75	0.59 (0.25)	0.80 (0.64)	.03
Trip distance						
1 to 5 Miles	62.96 (21.07)	67.39 (21.78)	.31	0.00 (0.04)	−0.18 (0.07)	.03
5 to 10 Miles	26.56 (5.94)	27.41 (6.13)	.47	0.01 (0.03)	0.02 (0.06)	.84
10 to 20 Miles	32.56 (5.79)	34.11 (5.99)	.21	0.00 (0.02)	−0.01 (0.04)	.93
Hours driven	52.57 (9.56)	58.17 (9.89)	.01	0.01 (0.05)	−0.12 (0.08)	.37
Trip time, min	19.39 (3.97)	21.64 (4.12)	.02	−0.01 (0.02)	−0.06 (0.03)	.19
Days driven per month	28.86 (3.68)	29.72 (3.80)	.24	−0.02 (0.02)	−0.08 (0.03)	.01
Radius of gyration	11.91 (45.18)	15.95 (46.99)	.75	0.67 (0.54)	3.11 (1.04)	.01
Maximum distance from home, km	94.21 (233.07)	49.36 (241.39)	.52	8.01 (3.85)	31.12 (7.39)	<.001
Maximum distance driven, km	69.99 (210.84)	9.00 (218.22)	.35	9.25 (3.87)	32.27 (7.42)	<.001
Random entropy	5.15 (0.60)	5.43 (0.62)	.03	−0.02 (0.00)	0.01 (0.01)	<.001
Destinations visited	43.28 (12.52)	48.01 (12.99)	.08	−0.27 (0.05)	0.34 (0.10)	<.001

## Discussion

The projected increase in the geriatric population will continue the current heavy reliance on personal driving for community mobility, sustaining employment, community participation, self-reliance, and aging in place. We hypothesized that drivers with MDD would experience significant changes in naturalistic driving behavior over time, particularly in more risky behaviors like speeding and hard braking, visiting fewer destinations, and having a smaller number of trips compared with those who did not have depression. This hypothesis was largely supported by analysis of the naturalistic driving data, which suggested that older drivers with MDD had a distinct pattern compared with the control group. Based on intercepts, participants with MDD demonstrated a propensity toward riskier driving habits with a higher frequency of speeding events and spending more time on the road compared with those without MDD. Additionally, during a mean of 1.1 years of follow-up, individuals with MDD were associated with more hard braking and hard cornering events per trip, drove greater distances from their homes, visited more unique destinations, and had a higher degree of random entropy and radius of gyration. Most importantly, our findings demonstrate that MDD (a common and treatable illness in older adults) was associated with an increase in both the amount and magnitude of risky driving behaviors over time.

Self-regulation is a common strategy for older drivers to modulate the risk of a crash in response to declining vision, poor general health, increasing frailty, and cognitive decline.^30,31^ Adjustments include minimizing night trips or venturing out during inclement weather (eg, rain or snow), not using unfamiliar roads, avoiding high-traffic situations, and driving more slowly than the posted speed limit.^32^ These activity constraints may be intentional or subconscious changes to preserve safety by reducing the need to engage in behaviors such as hard braking in response to a stimulus like high traffic in the environment or inattention to the road.^33^ Our findings suggest older drivers with MDD do not engage in safer behaviors over time, consistent with prior evidence that depression may impair insight and awareness^34^ of slowly declining motor, sensory, or cognitive processes (eg, attentional control)^35^ relevant for assessing safety. Conversely, those without MDD showed a decline in random entropy, suggesting more of a predictable driving pattern over time reflective of a set routine and likely awareness of their functional abilities.

Despite these nuanced differences in driving behavior, both groups maintained similar levels of overall driving activity during the study period, as evidenced by comparable intercepts and slopes in average time spent per trip, hours driven per month, and various trip lengths based on their behaviors. This finding suggests that while depression may influence specific aspects of driving behavior, it does not necessarily lead to reduced overall driving engagement among older adults. However, this in vivo examination of daily behavior reveals that older adults with MDD had more trips with random entropy, demonstrating greater unpredictability in their driving patterns, more destinations visited, larger distances driven from their homes, and higher frequency in hard braking. While these behaviors may not portend an imminent crash risk, the combination of these increases the likelihood of a near miss and crashes.

Antidepressants are an established, potentially driver-impairing medication and are known to impact driving performance on a road test.^36^ A recent study^37^ of 2951 older drivers using 12 months of naturalistic driving found that self-reported depression alone did not predict changes in driving behaviors, but antidepressants were associated with 1.22 higher odds of hard braking events. A recent meta-analysis^38^ found that antidepressant pharmacotherapy of late-life depression found improvements in memory and learning domains. Our results extend the current literature by demonstrating that MDD alone was associated with risky driving behavior and other spatial metrics of organization (eg, entropy). The addition of an antidepressant composite and a separate model with total medications did not alter the primary results, demonstrating that MDD had a unique association with driving over time.

The presence of multiple comorbidities among individuals with MDD underscores the complexity of managing depression in this population and suggests the need for comprehensive care approaches that address both mental and physical health. This finding is consistent with previous research highlighting the association between depression and physical health ailments in older adults.^39^ Late-life depression and dementia share common cognitive features such as performance deficits in reaction time, divided attention, impaired executive function, and lapses in short-term memory.^14^ While participants with MDD had a higher CDR-SB and global rating on the CDR, this clinical finding should not be interpreted as participants with MDD having more cognitive symptoms indicative of dementia, thus leading to worse driving. MDD alone can increase scores on the CDR-SB independent of cognitive impairment.^40^ Neuropsychological performance across different domains and the cognitive composite score suggested no group difference in cognitive functioning. This is crucial since driving is irreducible to singular cognitive domains under a controlled task or test. Its complexity and dynamic nature in real-time provide a valuable window demonstrating the association between depression and strategic, tactical, and operational levels of driving.

This study employed a rigorous approach to recruiting a psychiatric population of older adults with MDD and compared them with older adults without MDD using IPTW,^29^ a methodologically rigorous technique to interrogate the association between MDD and driving. Naturalistic driving behavior was collected daily in the participant’s own vehicle and driving environment for a prolonged duration. Additionally, the use of driver-impairing medications was adjusted in post hoc analyses, yet the results remained unchanged.

There is a pressing need for targeted interventions to manage and mitigate the driving risks associated with late-life depression. Regular screening for depression and cognitive impairments in older drivers, coupled with assessing driving fitness and tailored driving safety programs and support systems, can help enhance road safety and maintain the independence of older adults.

### Limitations

This study had limitations. We examined MDD at baseline and did not examine how symptoms may have changed over follow-up; likewise, other psychiatric conditions that covary with MDD were not adjusted for in the analyses. It is well understood that the CDR as a criterion standard struggles when separating depression from dementia, but it remains our most effective tool when screening participants with preexisting memory impairment unrelated to depression. Finally, driving did not interrogate specific behavioral aspects like situational factors related to their home environment or driver perceptions. Future research should explore the underlying mechanisms linking depression to longitudinal changes in driving behavior and evaluate the effectiveness of interventions aimed at promoting safe driving behaviors among older adults with MDD.

## Conclusions

In this study, older drivers with MDD had more hard braking and hard cornering events and drove further and to more unique destinations than their peers without depression. Identifying distinct patterns of driving behavior associated with depression can inform targeted interventions like cognitive retraining or driver rehabilitation by occupational therapists that may support safe mobility and enhance the well-being of older drivers with MDD. Additionally, psychiatrists should be aware of the potential impact of medications on driving abilities and consider these factors when prescribing treatments for older adults with depression. By doing so, we can better support the aging population in maintaining safe driving practices and improving their overall quality of life.

## Notes

Supplement 1. **eTable 1**. Medications Included in Analysis **eTable 2**. Driving Behavior Outcomes Across Drivers With and Without Major Depressive Disorder, Where Each Variable Represents the Mean Number per Trip or Summary per Month (Addition of Total Medication Use) **eTable 3**. Description of the GPS-Based Driving Features

Supplement 2.
Nonauthor Collaborators

Supplement 3.
Data Sharing Statement
