# Supplementary material for: Major Depressive Disorder and Driving Behavior Among Older Adults
Source: JAMA Netw Open. 2024 Dec 30;7(12):e2452038. doi: 10.1001/jamanetworkopen.2024.52038 (PMC11686415; doi:10.1001/jamanetworkopen.2024.52038)
Supplement: Supplement 1. — eTable 1. Medications Included in Analysis eTable 2. Driving Behavior Outcomes Across Drivers With and Without Major Depressive Disorder, Where Each Variable Represents the Mean Number per Trip or Summary per Month (Addition of Total Medication Use) eTable 3. Description of the GPS-Based Driving Features [file jamanetwopen-e2452038-s001.pdf]

# Supplemental Online Content

Babulal GM, Chen L, Trani J-F, et al. Major depressive disorder and driving behavior among older adults. *JAMA Netw Open*. 2024;7(12):e2452038. doi:10.1001/jamanetworkopen.2024.52038

**eTable 1**. Medications Included in Analysis

**eTable 2**. Driving Behavior Outcomes Across Drivers With and Without Major Depressive Disorder, Where Each Variable Represents the Mean Number per Trip or Summary per Month (Addition of Total Medication Use)

**eTable 3**. Description of the GPS-Based Driving Features

This supplemental material has been provided by the authors to give readers additional information about their work.

© 2024 Babulal GM et al. *JAMA Network Open.*

| **eTable 1**. Medications Included in Analysis | |
|---|---|
| **Categories** | **Class (if available) Medication** |
| Selective serotonin reuptake inhibitor/Serotonin–norepinephrine reuptake inhibitor | citalopram, desvenlafaxine, duloxetine, escitalopram, fluoxetine, paroxetine, sertraline, venlafaxine, vilazodone |
| Monoamine oxidase inhibitor | phenelzine, selegiline |
| Tricyclic antidepressant | amitriptyline, doxepin, imipramine, nortriptyline |
| Antipsychotics | aripiprazole, cariprazine, prochlorperazine, quetiapine |
| Benzodiazepines | alprazolam, chlordiazepoxide, diazepam, lorazepam, temazepam, triazolam |
| Anticonvulsants | carbamazepine, clonazepam, gabapentin, lamotrigine, levetiracetam, pregabalin, primidone, topiramate |
| Opioids | acetaminophen/codeine, acetaminophen/hydrocodone, acetaminophen/oxycodone, acetaminophen/propoxyphene, acetylsalicylic acid/caffeine/propoxyphene, codeine/guaifenesin/pseudoephedrine, hydrocodone, morphine, oxycodone, propoxyphene, tramadol |
| Nonsteroidal anti-inflammatory drug /Acetaminophen | acetaminophen, acetaminophen/acetylsalicylic acid/caffeine, acetaminophen/butalbital/caffeine, acetaminophen/caffeine, acetaminophen/codeine, acetaminophen/diphenhydramine, acetaminophen/hydrocodone, acetaminophen/phenyltoloxamine, acetaminophen/propoxyphene, acetaminophen/pseudoephedrine, acetylsalicylic acid/anhydrous citric acid/sodium bicarbonate, acetylsalicylic acid/caffeine/propoxyphene, acetylsalicylic acid/dipyridamole, aspirin, celecoxib, diclofenac, etodolac, ibuprofen, indomethacin, ketorolac, meloxicam, nabumetone, naproxen, piroxicam, rofecoxib |
| Central nervous system drugs | acetaminophen, acetaminophen/acetylsalicylic acid/caffeine, acetaminophen/butalbital/caffeine, acetaminophen/caffeine, acetaminophen/codeine, acetaminophen/diphenhydramine, acetaminophen/hydrocodone, acetaminophen/oxycodone, acetaminophen/phenyltoloxamine, acetaminophen/propoxyphene, acetaminophen/pseudoephedrine, acetylsalicylic acid/anhydrous citric acid/sodium bicarbonate, acetylsalicylic acid/caffeine/propoxyphene, acetylsalicylic acid/dipyridamole, almotriptan, alprazolam, amitriptyline, amphetamine, aripiprazole, armodafinil, aspirin, atropine/hyoscyamine/phenobarbital/scopolamine, bupropion, buspirone, butalbital, carbamazepine, carbidopa/levodopa, cariprazine, celecoxib, chlordiazepoxide, citalopram, clonazepam, codeine/guaifenesin/pseudoephedrine, cyclohexane, desvenlafaxine, |

© 2024 Babulal GM et al. *JAMA Network Open.*

| | |
|---|---|
| | dextromethorphan/quinidine, diazepam, diclofenac, doxepin, duloxetine, eletriptan, escitalopram, eszopiclone, etodolac, fluoxetine, gabapentin, hydrocodone, ibuprofen, imipramine, indomethacin, ketorolac, lamotrigine, levetiracetam, lidocaine, lisdexamfetamine, lorazepam, meloxicam, memantine, methylphenidate, milnacipran, mirtazapine, modafinil, morphine, nabumetone, naltrexone, naproxen, nefazadone, nortriptyline, oxycodone, paroxetine, phenelzine, phentermine, piroxicam, pramipexole, pregabalin, primidone, prochlorperazine, procyclidine, propoxyphene, quetiapine, rofecoxib, ropinirole, secobarbital, selegiline, sertraline, sumatriptan, suvorexant, temazepam, topiramate, tramadol, trazodone, triazolam, tryptophan, venlafaxine, vilazodone, zaleplon, zolpidem, zolmitriptan |
| Any Antidepressant | amitriptyline, bupropion, citalopram, desvenlafaxine, doxepin, duloxetine, escitalopram, fluoxetine, imipramine, mirtazapine, nefazodone, nortriptyline, paroxetine, phenelzine, sertraline, trazodone, venlafaxine, vilazodone |
| Total composite | A count of all drugs listed above |
| Notes: All brand names are converted to generic names | |

© 2024 Babulal GM et al. *JAMA Network Open.*

**eTable 2**. Driving Behavior Outcomes Across Drivers With and Without Major Depressive Disorder, Where Each Variable Represents the Mean Number per Trip or Summary per Month (Addition of Total Medication Use)

| | Y-intercept | | | | | Slope | | | | |
|---|---|---|---|---|---|---|---|---|---|---|
| **Naturalistic driving** | **Controls** | | **Cases** | | | **Controls** | | **Cases** | | |
| | Mean | SE | Mean | SE | *p* | Mean | SE | Mean | SE | *p* |
| Hardcore braking | 2.28E-3 | 4.30E-3 | 1.38E-3 | 4.40E-3 | 0.291 | 7.70E-5 | 3.00E-5 | 3.06E-4 | 7.10E-5 | **<.001** |
| Sudden acceleration | 0.01 | 0.03 | 0.01 | 0.03 | 0.168 | 1.47E-4 | 1.22E-4 | 2.16E-4 | 2.15E-4 | 0.295 |
| Speeding | 26.54 | 32.27 | 48.63 | 33.65 | **0.013** | -0.17 | 0.14 | -0.16 | 0.27 | 0.408 |
| Hard cornering | 33.75 | 20.68 | 33.47 | 21.73 | 0.964 | 0.59 | 0.25 | 0.78 | 0.65 | **0.035** |
| | | | | | | | | | | |
| Trips 1 to 5 miles | 65.92 | 20.95 | 70.55 | 21.57 | 0.275 | 0.00 | 0.04 | -0.18 | 0.06 | **0.018** |
| Trips 5 to 10 miles | 26.69 | 5.92 | 27.38 | 6.08 | 0.541 | 0.01 | 0.03 | 0.02 | 0.06 | 0.841 |
| Trips 10 to 20 miles | 32.12 | 5.87 | 33.04 | 6.04 | 0.434 | 0.00 | 0.02 | -0.01 | 0.04 | 0.933 |
| Hours driven | 53.07 | 9.60 | 58.23 | 9.90 | **0.014** | 0.01 | 0.05 | -0.12 | 0.08 | 0.371 |
| Mean trip time mins. | 19.19 | 3.98 | 21.37 | 4.11 | **0.023** | -0.01 | 0.02 | -0.06 | 0.03 | 0.184 |
| Days driven per month | 29.60 | 3.68 | 30.46 | 3.78 | 0.227 | -0.02 | 0.02 | -0.08 | 0.03 | **0.010** |
| | | | | | | | | | | |
| Radius of gyration | 16.99 | 44.94 | 23.42 | 46.57 | 0.598 | 0.67 | 0.54 | 3.12 | 1.04 | **0.006** |
| Max dist. from home | 127.78 | 230.08 | 98.94 | 237.35 | 0.667 | 8.07 | 3.84 | 31.20 | 7.37 | **<.001** |
| Max dist. driven | 95.31 | 208.41 | 46.74 | 214.86 | 0.446 | 9.29 | 3.86 | 32.34 | 7.40 | **<.001** |
| Random entropy | 5.21 | 0.61 | 5.50 | 0.64 | **0.024** | -0.02 | 0.00 | 0.01 | 0.01 | **<.001** |
| Destinations visited | 43.73 | 12.82 | 48.13 | 13.26 | 0.098 | -0.27 | 0.05 | 0.34 | 0.10 | **<.001** |

© 2024 Babulal GM et al. *JAMA Network Open.*

**eTable 3**. **Description of the GPS-Based Driving Features**

| | Indicator (aggregated monthly) | Units | Description |
|---|---|---|---|
| 1 | Number of Hardcore Braking Events | Count | Total number of detected events where a vehicle moving at a speed greater than 18.64 miles per hour (MPH) (30 kilometer per hour (KPH)) shows a decrease in speed exceeding a rate of 11.81 MPH (19 KPH) per second. |
| 2 | Rate of Hardcore Braking | Events per mile | Calculated by the total number of hardcore braking events divided by total distance (miles) traveled in each month. |
| 3 | Number of Hard Cornering Events | Count | Total number of detected events where the lateral acceleration of the vehicle exceeds a threshold of 0.4 g-force (g), sustained for at least 12 counts (0.5s)within a sliding window size of 36 (1.5s). |
| 4 | Rate of Hard Cornering | Events per mile | Calculated by the total number of hard cornering events divided by total distance traveled in each month. |
| 5 | Number of Days Driven | Count | Total number of days per month when a vehicle completes a trip (ignition on / ignition off cycle) with a trip distance greater than 0 miles. |
| 6 | Number of Trips less than 1 mile | Count | Total number of trips with a distance smaller than 1 mile in each month. |
| 7 | Number of Trips between 1 mile to 5 miles | Count | Total number of trips with a distance between 1 and 5 miles in each month. |
| 8 | Random Entropy | Bits | $$E_{rand}(u) = log_2(N_u)$$ $N_u$ is the number of distinct locations visited by $u$, capturing the degree of predictability of $u$'s whereabouts if each location is visited with equal probability[1]. |
| 9 | Radius of Gyration | Miles | Compute the radii of gyration of a set of individuals in a TrajDataFrame. The radius of gyration of an individual is defined as: $$r_g(u) = \sqrt{\frac{1}{n_u}\sum_{i=1}^{n_u} dist(r_i(u) - r_{cm}(u))^2}$$ where $r_i(u)$ represents the $n_u$ positions recorded for $u$, and $r_{cm}(u)$ is the center of mass of $u$'s trajectory. In mobility analysis, the radius of gyration indicates the characteristic distance travelled by $u$ [2]. |
| 10 | Maximum Distance from Home | Miles | Maximum distance travelled from home in each month. Compute the maximum distance traveled from their home location by a set of individuals in a TrajDataFrame. The maximum distance from home $dh_{\max}(u)$ of an individual is defined as: $$dh_{\max}(u) = \max_{1 \le i < j < n_u} dist(r_i, h(u))$$ |

© 2024 Babulal GM et al. *JAMA Network Open.*

| | | | |
|---|---|---|---|
| | | | where $N_u$ is the number of points recorded for $u$, $r_i$ is a location visited by $u$ described as a pair, $h(u)$ is the home location of $u$, and $dist$ is the geographic distance between two points[3]. |
| 11 | Maximum of Distance | Miles | Maximum distance traveled in each month. |
| 12 | Number of unique destinations | Count | Total number of unique destinations visited in each month[4]. |

© 2024 Babulal GM et al. *JAMA Network Open.*
