# Supplementary material for: Major Depressive Disorder and Driving Behavior Among Older Adults
Source: JAMA Netw Open. 2024 Dec 30;7(12):e2452038. doi: 10.1001/jamanetworkopen.2024.52038 (PMC11686415; doi:10.1001/jamanetworkopen.2024.52038)
Supplement: Supplement 2. — Nonauthor Collaborators [file jamanetwopen-e2452038-s002.pdf]

Supplemental Online Content: Nonauthor Collaborators
*First name, last name, and suffix (if applicable) are required and will appear in PubMed.

***Group Name(s): The DRIVES Project**

***First Name and Middle Initial(s)**	***Last Name**	***Suffix (eg, Jr, III)**	Academic Degrees	Institution	Location (city, state/province, country)	Role or Contribution, eg, chair, principal investigator	Group (if more than 1 Group listed in the byline) and/or Subgroup (eg, Steering Committee)
Kaylin	Taylor		BA, MS	Washington University School of Medicine	St. Louis, Missouri, USA	Clinical Research Coordinator	The DRIVES Project
Maeve	Intagliata		BS, MS	Washington University School of Medicine	St. Louis, Missouri, USA	Clinical Research Coordinator	The DRIVES Project
Nikitha	Damera		BA	Washington University School of Medicine	St. Louis, Missouri, USA	Clinical Research Coordinator	The DRIVES Project
Carson	Woodfin		BS	Washington University School of Medicine	St. Louis, Missouri, USA	Clinical Research Coordinator	The DRIVES Project
Ann M.	Johnson		HS	Washington University School of Medicine	St. Louis, Missouri, USA	Clinical Research Coordinator	The DRIVES Project
Matthew	Blake		BA, MS	Washington University School of Medicine	St. Louis, Missouri, USA	Bioinformaticist	The DRIVES Project
Chen	Chen		BA, MSW, MPH	Washington University School of Medicine	St. Louis, Missouri, USA	Statistical Data Analyst	The DRIVES Project
