# Supplementary material for: Major Depressive Disorder and Driving Behavior Among Older Adults
Source: JAMA Netw Open. 2024 Dec 30;7(12):e2452038. doi: 10.1001/jamanetworkopen.2024.52038 (PMC11686415; doi:10.1001/jamanetworkopen.2024.52038)
Supplement: Supplement 3. — Data Sharing Statement [file jamanetwopen-e2452038-s003.pdf]

# Data Sharing Statement

Babulal. Major Depressive Disorder and Driving Behavior Among Older Adults. *JAMA Netw Open*. Published December 26, 2024. doi:10.1001/jamanetworkopen.2024.52038

**Data**
**Data available:** No

**Additional Information**
**Explanation for why data not available:** There are sensitive identifying data (e.g., latitude, longitude) that could be reversed engineered to identify participants and where they live.
